# Antimicrobial susceptibility of travel-related *Salmonella enterica* serovar Typhi isolates detected in Switzerland (2002–2013) and molecular characterization of quinolone resistant isolates

**DOI:** 10.1186/s12879-015-0948-2

**Published:** 2015-05-12

**Authors:** Magdalena Nüesch-Inderbinen, Helga Abgottspon, Grethe Sägesser, Nicole Cernela, Roger Stephan

**Affiliations:** Institute for Food Safety and Hygiene, National Centre for Enteropathogenic Bacteria and Listeria, Vetsuisse Faculty University of Zurich, Winterthurerstr 272, CH-8057 Zurich, Switzerland

**Keywords:** Typhoid fever, MDR, Nalidixic acid, Ciprofloxacin, GyrA, ParC, PMQR

## Abstract

**Background:**

Typhoid fever is an acute, invasive, and potentially fatal systemic infection caused by *Salmonella enterica* subspecies *enterica* serotype Typhi (*S.* Typhi). Drug resistance to antimicrobials such as ciprofloxacin is emerging in developing countries, threatening the efficacy of treatment of patients in endemic regions as well as of travellers returning from these countries.

**Methods:**

We compared the antimicrobial resistance profiles of 192 *S*. Typhi isolated from patients over a time span of twelve years. Susceptibility testing was done by the disk diffusion method. A representative selection of isolates (n = 41) was screened by PCR for mutations in the quinolone resistance-determining regions (QRDRs) of the *gyrA* and *parC* genes and all 192 isolates were screened for plasmid-mediated quinolone resistance (PMQR) genes. Multilocus sequence typing (MLST) was used to investigate the sequence type of isolates from patients with a known history of international travel.

**Results:**

Resistance rates for nalidixic acid increased from 20 % to 66.7 % between 2002 and 2013. Resistance to ciprofloxacin was detected in 55.6 % of the isolates by 2013. Ciprofloxacin resistance was predominantly associated with the triple substitutions Ser83 → Phe and Asp87 → Asn in GyrA and Ser80 → Ile in ParC. The plasmid-mediated resistance gene *qnrS1* was detected in two isolates. Sequence type ST1 was associated with the Indian subcontinent, while ST2 was distributed internationally. Multidrug resistance was noted for 11.5 % of the isolates.

**Conclusions:**

Fluoroquinolone resistant *S*. Typhi constitute a serious public health concern in endemic areas as well as in industrialized countries. Increased surveillance of global patterns of antimicrobial resistance is necessary and the control of resistant strains is of the utmost importance to maintain treatment options.

**Electronic supplementary material:**

The online version of this article (doi:10.1186/s12879-015-0948-2) contains supplementary material, which is available to authorized users.

## Background

*Salmonella enterica* subspecies *enterica* serovar Typhi (*S.* Typhi) is a human-specific etiological agent of an acute, invasive, and potentially fatal systemic infection also known as typhoid fever [1]. The disease is a serious public health problem in developing countries with the Indian subcontinent (India, Pakistan, Afghanistan, Nepal and Bangladesh) representing a geographic area of intense disease activity [2]. In industrialized countries typhoid fever is rare and mainly associated with travel to high-risk destinations [3, 4]. In the 1990ies, antimicrobial therapy became problematic in endemic areas due to multi-drug resistant (MDR) *S*. Typhi, rendering chloramphenicol, trimethoprim and ampicillin, which were the drugs used as first-line treatment ineffective. Currently, fluoroquinolones such as ciprofloxacin are recommended by the WHO [5], as they are the most effective orally active, inexpensive and well-tolerated drugs for the treatment of typhoid fever [6]. Hence, the worrisome signs of increasing resistance to this class of antimicrobials represent a challenge for effective control and management of typhoid fever, especially in endemic countries. Resistance is mainly due to the accumulation of mutations in the quinolone resistance-determining regions (QRDRs) of DNA gyrase and topoisomerase IV genes (target site alterations). Additionally, plasmid-mediated quinolone resistance (PMQR) confer a form of reduced susceptibility which is thought to promote the accumulation of the target site mutations [7]. PMQR include the Qnr families of pentapeptide repeat proteins encoded by *qnrA, qnrB, qnrC qnrD* and *qnrS*, [8, 9] and [10]. The majority of mutations in quinolone resistant *S*. Typhi have been detected within the quinolone resistance-determining region (QRDR) of the DNA gyrase gene *gyrA* and in the topoisomerase gene *parC* [11]. Primary mutations in target site genes generate resistance to the quinolone antimicrobial nalidixic acid, making it useful as a surrogate marker of decreased susceptibility to fluoroquinolones [12], as resistance to nalidixic acid correlates with decreased susceptibility to ciprofloxacin and hence to potential treatment failure [13–15].

The aim of this study was to describe the antimicrobial susceptibility profiles of *S*. Typhi isolated from patients over a time span of twelve years. In a selection of isolates, the presence of point mutations in the genes *gyrA* and *parC* that encode the quinolone targets was determined. The clonal relationship of isolates known to be associated with international travel was assessed.

## Methods

### Clinical isolates

Between 2002 and 2013, 203 *S.* Typhi isolates were sent to the National Centre for Enteropathogenic Bacteria and Listeria (NENT), Switzerland, for final serological identification and strain collection. All samples had been submitted by hospitals or primary diagnostic laboratories located in Switzerland. The number of collected strains varied from 10 to 24 per annum, with a median of 16. Patients’ data was anonymized. Studies performed with such strains had previously been declared unobjectionable by the ethics commission of the Canton of Zürich, Switzerland (declaration KEK Stv.-Nr. 19/12). For the study, only one isolate per patient was selected. Isolates from family members were considered separate samples (10 samples from 4 families). Of the 203 collected isolates, 192 met these criteria for further analysis.

### Antimicrobial susceptibility tests

Susceptibility testing was performed for the 192 isolates using the Kirby Bauer disk diffusion method and the antimicrobials nalidixic acid, ciprofloxacin, ampicillin, amoxicillin/clavulanic acid, cephalotin, cefotaxime, gentamicin, kanamycin, streptomycin, tetracycline, sulfamethoxazole, trimethoprim and chloramphenicol (Becton Dickinson, Heidelberg, Germany). Results were interpreted according to the Clinical and Laboratory Standards Institute (CLSI) [16]. Hereby, breakpoints for ciprofloxacin are ≥31 mm (susceptible), 21–30 mm (intermediate) and ≤20 mm (resistant).

Multidrug-resistance (MDR) was defined as resistance to conventional antityphoid drugs ampicillin, trimethoprim-sulfamethoxazole and chloramphenicol (13).

### Molecular characterization of quinolone resistance genes

DNA was extracted using a standard heat lysis protocol. In brief, 5 bacterial colonies were resuspended in 450 μl lysis buffer containing 0.1 M Tris pH 8.5, 0.05 % Tween-20 and 240 μg/ml proteinase K. Samples were incubated at 60 °C for 60′, followed by 97 °C for 15′. Lysates were centrifuged for 10″ at 13,200 rpm and 5 μl of the supernatant were used for PCR.

A subset of 41 isolates eliciting various resistance patterns and representing different years of isolation was subjected to PCR-based detection of mutations in the quinolone resistance-determining regions (QRDRs) of the *gyrA* and *parC* genes, using amplification primers described previously [17]. All 192 isolates were screened for plasmid-mediated quinolone resistance (PMQR) genes using primers described previously for *qnrA* [17, 18], *qnrB* [19], *qnrC* [20], *qnrD* [21], and *qnrS* [17]. Custom sequencing was performed by Microsynth (Balgach, Switzerland) and nucleotide sequences were analysed with CLC Main Workbench 6.6.1. For database searches the BLASTN program of NCBI (http://www.ncbi.nlm.nih.gov/blast/) was used.

### Multilocus sequence typing

Multilocus sequence typing (MLST) [22] was used to investigate the sequence type of 26 isolates from patients with a known history of international travel. These isolates were randomly selected from 83 travel-associated isolates. Internal fragments of the seven housekeeping genes *aroC*, *dnaN*, *hemD*, *hisD*, *purE*, *sucA* and *thrA* were amplified by PCR. Sequencing of the amplification products was performed by Microsynth (Balgach). Sequence types (ST) were assigned in accordance with the *Salmonella enterica* MLST Database http://mlst.ucc.ie/mlst/dbs/Senterica.

## Results and discussion

A detailed overview of the 192 *S*. Typhi isolates analysed in this study, together with anamnestic data and any travel information of the patients is given in Additional file 1: Table S1. The majority of the strains (96 %) were isolated from blood or stool. Patient median age was 26 years (range <1–74 years), ninety-five (49.5 %) were female and 21.4 % were children (<16 years). Information on the travel background was availabe for 83 patients. Thereof, the three main countries involved were India with 41 patients (49.4 %), Pakistan with 7 (8.4 %) and Bangladesh with 4 (4.8 %) patients.

The rate of full susceptibility of *S*. Typhi to the antimicrobials tested in this study declined from 80 % in 2002 to 28 % in 2013. This decrease was mainly due to the emergence of isolates with resistance to nalidixic acid, with resistance rates increasing from 20 to 66.7 % during the same period. Ciprofloxacin resistance rose from 0 to 55.6 %, albeit variably (Table 1). Multidrug-resistance (MDR), and especially resistance to former drugs of first choice ampicillin, trimethoprim-sulfamethoxazole and chloramphenicol [13], was observed variably (0–25 %, Table 1). Resistance to ampicillin-clavulanic acid and cephalotin was found in one and 8 isolates, respectively, while streptomycin and tetracycline resistance was detected in 24 (12.5 %) and 15 (7.8 %) isolates, respectively (Additional file 1: Table S1).

**Table 1:** Number and proportion (%) of nalidixic acid-, ciprofloxacin- and multidrug-resistant *Salmonella* Typhi isolated between 2002 and 2013

		Resistance pattern		
Total no. of isolates		NAL	CIP	MDR^a^
	Year	no. (%)	no. (%)	no. (%)
10	2002	2 (20)	0 (0)	0 (0)
16	2003	6 (37.5)	0 (0)	2 (12.5)
13	2004	5 (38.5)	1 (7.7)	2 (15.4)
16	2005	5 (31.3)	3 (18.8)	1 (6.3)
16	2006	9 (56.3)	3 (18.8)	0 (0)
18	2007	15 (83.3)	5 (27.8)	3 (16.7)
16	2008	9 (56.3)	3 (18.8)	3 (18.8)
18	2009	14 (77.8)	1 (5.6)	1 (5.6)
24	2010	20 (83.3)	6 (25)	4 (16.7)
15	2011	9 (60)	4 (26.7)	3 (20)
12	2012	7 (58.3)	3 (25)	3 (25)
18	2013	12 (66.7)	10 (55.6)	0 (0)
Total				
192		113 (58.9)	39 (20.3)	22 (11.5)

Abbreviations: NAL, nalidixic acid; CIP, ciprofloxacin; MDR, multidrug resistant

^a^Multidrug resistance is defined as resistance to ampicillin, trimethoprim/sulfamethoxazole and chloramphenicol (13)

In total, 113 (58.9 %) of the isolates were resistant to nalidixic acid. Of the 36 analysed nalidixic acid-resistant isolates, 35 (97 %) contained a substitution in Ser83 of GyrA, whereby 27 (75 %) contained the amino acid substitution Ser83 → Phe and the remaining 8 carried the mutation Ser83 → Tyr (Table 2). Only one nalidixic acid and ciprofloxacin resistant isolate (N10-1839, see Additional file 1: Table S1) tested negative for substitutions in GyrA. This clearly illustrates the predominance of *gyrA* mutations in quinolone resistant *S.* Typhi.

**Table 2:** Mutations in QRDR of GyrA and ParC observed in 35 nalidixic acid- and/or ciprofloxacin-resistant *S*. Typhi isolated between 2002 and 2013 and corresponding zone diameters obtained by the disk diffusion test for ciprofloxacin

	Mutation				
No. of isolates (%)	GyrA	ParC	No. of CIP resistant isolates (zone diameters [mm])^a^	Years of isolation	Identified associated country (no. of isolates)
8 (22.9)	Ser83 → Tyr		4 (20)	2002–2009	India (2), Bangladesh (1)
18 (51.4)	Ser83 → Phe		5 (19–21)	2003–2011	India (2), Pakistan (3), Bangladesh (1), Cambodia (1)
1 (2.9)	Ser83 → Phe	Glu84 → Gly	1 (14)	2006	India (1)
1 (2.9)	Ser83 → Phe	Glu84 → Lys	0 (21)	2008	India (1)
7 (20)	Ser83 → Phe / Asp87 → Asn	Ser80 → Ile	7 (6–11)	2010–2013	India (2), Nepal (1)

Abbreviation: CIP, ciprofloxacin

^a^Breakpoints for ciprofloxacin according to CLSI guidelines (16): susceptible, ≥30 mm; intermediate, 21–30 mm; resistant, ≤20 mm

In total, 39 (20.3 %) of the isolates were resistant to ciprofloxacin. The first ciprofloxacin-resistant strain from the collection described in this study was isolated in 2004 from a traveller from Cambodia (isolate 1736–04) and was associated with a Ser83 → Phe in GyrA and a zone diameter of 19 mm in the disk diffusion test for ciprofloxacin (Additional file 1: Table S1 and Table 2). One ciprofloxacin-resistant isolate contained an additional Glu84 → Gly substitution in ParC (isolate 1715–06, see Additional file 1: Table S1). In the disk diffusion test, this substitution was associated with a zone diameter of 14 mm (Table 2). One further isolate (1751–08) contained a Glu84 → Lys substitution in ParC. This substitution has been described previously in two ciprofloxacin-resistant isolates from Bangladesh [23]. However, those isolates both contained the two additional substitutions Trp106 → Gly and Tyr128 → Asp in ParC. Since isolate 1751–08 was categorized as intermediately susceptible to ciprofloxacin (zone diameter 21 mm), the Glu84 → Lys substitution in ParC does not appear to be crucial for ciprofloxacin resistance.

Ciprofloxacin-resistant strains isolated since 2010 (n = 23) all contained the combination of Ser83 → Phe and Asp87 → Asn in GyrA as well as the Ser80 → Ile substitution in ParC and were associated with decreased zone diameters of 6–11 mm in the disk diffusion tests for ciprofloxacin (Table 2). This genotype appears to be becoming more frequent compared to previous reports [11]. Although no *gyrB* or *parE* gene mutations were analysed in this study, the data presented here indicate these particular substitutions in GyrA and ParC may be considered a major quinolone resistance mechanism in *S*. Typhi. Mutations in *gyrA* are recognized to be predominant in nalidixic acid resistant *S*. Typhi [24, 25]. Increased efflux pump-mediated mechanisms, which are observed in other fluoroquinolone-resistant *Salmonella* [26], have very recently been reported to be absent in *S*. Typhi [27], further highlighting the importance of target-site mutations. Furthermore, a recent study by Baker et al. [24] has shown that mutations in the target genes *gyrA* and *parC* correlate with an increase of intrinsic fitness in *S*. Typhi. This indicates that the quinolone resistance genotypes observed in our study may continue to circulate even in the absence of antimicrobial selective pressure.

The plasmid-mediated quinolone resistance gene *qnrS1* was found in a fully susceptible isolate from 2009 and in a ciprofloxacin-resistant isolate from 2010 (isolates N09-2035 and N10-2349, respectively, see Additional file 1: Table S1). No other PMQR genes were detected in any of the isolates. There are few studies that describe PMQR genes in *S*. Typhi and our findings indicate that they may play a minor role in quinolone resistance of *S*. Typhi. The *qnrS* gene has to our knowledge been reported once in an isolate from the Democratic Republic of the Congo [28], whereas in quinolone-resistant *S*. Typhi isolates from India the *qnrB* gene predominates [29].

On account of travel histories, 54 isolates (28 %) were demonstrably associated with the Indian subcontinent (Additional file 1: Table S1). However, this number is possibly understated, due to missing travel information for many of the patients. This highlights the need to improve travel history reporting within the healthcare system in order to get a more precise picture of the association of distinct strains with distinct regions worldwide. Multilocus sequence typing of *S*. Typhi is limited by the fact that it discriminates 2 sequence types only. Nevertheless, typing assigned 15 isolates to ST1, of which 12 (80 %) were associated with the Indian subcontinent. By contrast, the origins of the 14 isolates assigned to ST2 were more widely dispersed and included Southeast Asia (4 isolates; 28.6 %), Africa (3 isolates; 21.4 %), Mexico (4 isolates; 29 %) and South America (1 isolate; 7.1 %). This finding is consistent with observations of Dahiya et al. [30] and indicates that ST2 may be circulating internationally, while ST1 mainly persists in the Indian subcontinent.

## Conclusions

This study presents an overview over the development of antimicrobial resistance profiles of *S*. Typhi over the last 12 years and provides molecular characteristics of nalidixic acid- and ciprofloxacin-resistant *S*. Typhi isolated in that time span. Since fluoroquinolones are currently the mainstay of the treatment of typhoid fever, resistance in *S.* Typhi threatens to limit therapeutic options and constitutes a serious public health concern in endemic areas as well as in industrialized countries. Increased surveillance of global patterns of antimicrobial resistance is necessary and the control of resistant strains is of the utmost importance.

This study adds to the current knowledge about the global circulation of quinolone-resistant *S*. Typhi and their associated mechanisms of resistance.

### Ethics statement

The Zurich Cantonal Ethics Commission evaluated the present study, and declared it legally and ethically unobjectionable.

## Additional file

Additional file 1: Table S1. S. Typhi 2002–2013. Details of the 192 *Salmonella* Typhi isolated between 2002 and 2013 analysed in this study.
